# The fundamental beliefs held by individuals living with HIV and university students during the challenges posed by the second wave of the COVID-19 pandemic in Russia

**DOI:** 10.1192/j.eurpsy.2024.1056

**Published:** 2024-08-27

**Authors:** V. V. Titova, V. I. Rozhdestvenskiy, I. A. Gorkovaya, D. O. Ivanov, Y. S. Aleksandrovich

**Affiliations:** Department of Psychosomatics and Psychotherapy, Saint Petersburg State Pediatric Medical University, Saint Petersburg, Russian Federation

## Abstract

**Introduction:**

Baseline beliefs, as conceptualized by R. Janoff-Bulman in her cognitive theory of mental trauma, represent an inherent internal framework that shapes how individuals perceive and assess traumatic events. It is widely acknowledged that the pandemic has had a profound impact on the global economy and the living conditions of individuals. Consequently, it is reasonable to assume that during a pandemic, one’s ability to adapt to these altered circumstances is influenced by their foundational beliefs. Consequently, exploring these fundamental beliefs in two at-risk groups, namely university students and individuals with confirmed HIV, becomes a subject of significant interest.

**Objectives:**

This study aims to examine the fundamental beliefs of patients with HIV and university students in the context of the second wave of the new coronavirus pandemic in Russia.

**Methods:**

Data collection took place from January to July 2021 using a custom-developed Google form. The study involved 35 Russian university students majoring in humanities and 59 HIV-positive patients. We employed the WAS-37 methodology, adapted for use in Russia, to assess their baseline beliefs.

**Results:**

We found that on the scales “Fairness” (M = 21.00±3.73 - students, M = 20.53±4.63 - patients, p = 0.616), “Luck” (M = 31.74±5.06 vs M = 29.59±7.33, p = 0.129) and “Control beliefs” (M = 26.66±4.80 vs M = 27.12±4.42, p = 0.636) students did not differ from patients. Scores on the Environment Benevolence scale were higher in students (M = 35.46±7.33 vs M = 30.50±7.09, p = 0.002) and on the Self Image scale were higher in HIV patients (M = 26.63±6.97 vs M = 30.03±5.41, p = 0.010).

**Conclusions:**

During the latter stages of the COVID-19 pandemic in Russia, individuals living with HIV, when compared to students, tended to perceive the world around them as being more perilous and unfriendly, while simultaneously viewing themselves as possessing greater integrity. From our perspective, this latter observation could be interpreted as a means of self-defence against the perceived hostility of the external world. In such pandemic circumstances, it may be advisable to consider the use of supportive psychotherapy for individuals living with HIV.

**Disclosure of Interest:**

None Declared

